# Comparative Lipidomics of Azole Sensitive and Resistant Clinical Isolates of *Candida albicans* Reveals Unexpected Diversity in Molecular Lipid Imprints

**DOI:** 10.1371/journal.pone.0019266

**Published:** 2011-04-29

**Authors:** Ashutosh Singh, Rajendra Prasad

**Affiliations:** Membrane Biology Laboratory, School of Life Sciences, Jawaharlal Nehru University, New Delhi, India; Institute of Developmental Biology and Cancer Research, France

## Abstract

Although transcriptome and proteome approaches have been applied to determine the regulatory circuitry behind multidrug resistance (MDR) in *Candida*, its lipidome remains poorly characterized. Lipids do acclimatize to the development of MDR in *Candida*, but exactly how the acclimatization is achieved is poorly understood. In the present study, we have used a high-throughput mass spectrometry-based shotgun approach and analyzed the lipidome of genetically matched clinical azole-sensitive (AS) and -resistant (AR) isolates of *C. albicans*. By comparing the lipid profiling of matched isolates, we have identified major classes of lipids and determined more than 200 individual molecular lipid species among these major classes. The lipidome analysis has been statistically validated by principal component analysis. Although each AR isolate was similar with regard to displaying a high MIC to drugs, they had a distinct lipid imprint. There were some significant commonalities in the lipid profiles of these pairs, including molecular lipid species ranging from monounsaturated to polyunsaturated fatty acid-containing phosphoglycerides. Consistent fluctuation in phosphatidyl serine, mannosylinositolphosphorylceramides, and sterol esters levels indicated their compensatory role in maintaining lipid homeostasis among most AR isolates. Notably, overexpression of either CaCdr1p or CaMdr1p efflux pump proteins led to a different lipidomic response among AR isolates. This study clearly establishes the versatility of lipid metabolism in handling azole stress among various matched AR isolates. This comprehensive lipidomic approach will serve as a resource for assessing strategies aimed at disrupting the functions of *Candida* lipids, particularly the functional interactions between lipids and MDR determinants.

## Introduction

The incidences of *Candida* cells acquiring multidrug resistance (MDR) are common, which in turn hamper their successful chemotherapy [1]–[4]. *C. albicans* as well as non-albicans species have evolved a variety of mechanisms to develop MDR to common antifungals. Reduced intracellular accumulation of drugs (due to rapid efflux) is one of the most prominent mechanisms of resistance in *Candida* cells. Accordingly, it has been well documented by several groups that clinical azole resistant (AR) isolates of *C. albicans* display transcriptional activation of genes encoding ATP Binding Cassette (ABC) multidrug transporter proteins CaCdr1p or CaCdr2p or Major Facilitator Super family (MFS) efflux pump protein CaMdr1p [5]–[8].

Lipids, in addition to being structural and metabolic components of yeast cells, also appear to play an indirect role in the frequently observed MDR in *Candida*. For example, CaCdr1p shows selectivity towards membrane recruitment and prefers membrane raft micro-domains for its localization within plasma membrane [9]. It has already been demonstrated that there are close interactions between raft constituents such as ergosterol and sphingolipids (SLs), and disruption of these results in altered drug susceptibilities [10], [11]. Thus, any change in ergosterol composition by disruption of *ERG* genes, or change in SL composition by disruption of its biosynthetic genes leads to improper surface localization of CaCdr1p [9]. Interestingly, MFS transporter CaMdr1p shows no such selectivity towards raft lipid constituents and remains fully membrane localized and functional in cells where sphingolipid or ergosterol biosynthesis is compromised [9]. There are also instances where common regulation of MDR and lipid metabolism genes have been observed [12], [13]. Any changes in the status of membrane lipid phase and asymmetry also seem to affect azole resistance in *Candida* cells [14]. Taken together, MDR in *Candida* is closely linked to the status of membrane lipids, wherein the overall drug susceptibility of a cell appears to be an interplay of membrane lipid environment, drug diffusion and extrusion [15].

Earlier studies describing changes in lipid composition in azole resistant isolates provided limited information, particularly due to the lack of high throughput analytical tools [16]–[20] and the use of randomly collected AS and AR isolates of *Candida* [21], [22]. In the present study, we have utilized high throughput MS-based platform to get an insight into the dynamics of lipids in frequently encountered azole resistance in *C. albicans* cells. We have performed comprehensive lipid profiling and compared the lipidomes of genetically matched pairs of azole sensitive (AS) and resistant (AR) hospital isolates of *C. albicans* and evaluated if any changes in lipid imprints are typical to a drug-resistant phenotype. In our analysis, we focused on the contents of five major groups of lipids namely: phosphoglycerides (PGLs), SLs, sterol esters (SEs), di-acyl and tri-acyl glycerols (DAGs and TAGs respectively) and analyzed their molecular species. The PGL groups including phosphatidyl choline (PC), phosphatidyl ethanolamine (PE), phosphatidyl inositol (PI), phosphatidyl serine (PS), phosphatidyl glycerol (PG) and phosphatidyl acid (PA), and SL groups including ceramide (CER), inositolphosphorylceramide (IPC), mannosylinositolphosphorylceramide (MIPC), mannosyldiinositolphosphorylceramide (M(IP)_2_C) were analyzed. Less abundant lyso-lipids namely lysophophatidylcholine (LysoPC), lysophophatidylethanolamine (LysoPE) and lysophophatidylglycerol (LysoPG) were also detected. Using the combination of comparative lipidomics and its statistical validation, we individually identified over 200 molecular lipid species and evaluated the differences in lipids between the AS and AR pairs. The study shows that though each isolate is different in regard to its lipid profile, it does share a few commonalities with the other isolates, particularly at the level of molecular lipid species. This study provides a comprehensive picture of total lipidome in response to azole resistance in *Candida* cells.

## Materials and Methods

### Lipid standards

Synthetic lipids with FA compositions that are not found, or are of very low abundance in *Candida*, were used as internal standards. Lipid standards were obtained from Avanti Polar Lipids (Alabaster, AL).

### Strains, media and culture conditions

*C. albicans* strains used in this study are listed in Supplementary Table S1. *C. albicans* cells were kept on YPD plates and inoculated in YPD medium (1% yeast extract, 2% glucose, and 2% bactopeptone). The cells were diluted into 50 ml fresh medium at 0.1 OD at A_600_ (∼10^6^ cells/ml) and grown for 14 h until the cells reached exponential growth (∼2×10^8^ cells/ml). Three separate cultures of each *Candida* strain were used.

### Lipid Extraction

Lipids were extracted from *Candida* cells using a slight modification of the method of Bligh and Dyer [23]. Briefly, the *Candida* cells were harvested at exponential phase and were suspended in 10 ml methanol. 4 g glass beads (Glaperlon 0.40–0.60 mm) were added and the suspension was shaken in a cell disintegrator (B. Braun, Melsungen, Germany) four times for 30 sec with a gap of 30 sec between shakings. Approximately 20 ml chloroform was added to the suspension to give a ratio of 2∶1 of chloroform∶methanol (v/v). The suspension was stirred on a flat-bed stirrer at room temperature for 2 hrs and then filtered through Whatman No. 1 filter paper. The extract was then transferred to a separatory funnel and washed with 0.2 volumes of 0.9% NaCl to remove the non-lipid contaminants. The aqueous layer was aspirated and the solvent of the lipid-containing, lower organic layer was evaporated under N_2_. The lipids were stored at −80°C until analysis.

### ESI-MS/MS lipid profiling

#### Phosphoglyceride Quantification

An automated ESI-MS/MS approach was used. Data acquisition and analysis was carried out as described previously by Devaiah et al. and Singh et al. [24], [25] with minor modifications. The extracted dry lipid samples were dissolved in 1 ml chloroform. An aliquot of 2 to 8 µl of extract in chloroform was analyzed, with the exact amount depending upon the dry lipid weight of each sample. Precise amounts of internal standards, obtained and quantified as previously described by Welti et al. [26], were added in the following quantities (with some small variation in amounts in different batches of internal standards): 0.6 nmol di12:0-PC, 0.6 nmol di24:1-PC, 0.6 nmol 13∶0-LysoPC, 0.6 nmol 19∶0-LysoPC, 0.3 nmol di12:0-PE, 0.3 nmol di23:0-PE, 0.3 nmol 14∶0-LysoPE, 0.3 nmol 18∶0-LysoPE, 0.3 nmol di14:0-PG, 0.3 nmol di20:0(phytanoyl)-PG, 0.3 nmol 14∶0-LysoPG, 0.3 nmol 18∶0-LysoPG, 0.3 nmol di14:0-PA, 0.3 nmol di20:0(phytanoyl)-PA, 0.2 nmol di14:0-PS, 0.2 nmol di20:0(phytanoyl)-PS, 0.23 nmol 16∶0-18∶0-PI, 0.16 nmol di18:0-PI. The sample and internal standard mixture was combined with solvents, such that the ratio of chloroform/methanol/300 mM ammonium acetate in water was 300/665/35 (v/v/v) in a final volume of 1.4 ml. Each PGL class was quantified in comparison to the two internal standards of that class.

Unfractionated lipid extracts were directly introduced by continuous infusion into the ESI source on a triple quadrupole MS (API 4000, Applied Biosystems, Foster City, CA). Samples were introduced using an autosampler (LC Mini PAL, CTC Analytics AG, Zwingen, Switzerland) fitted with the required injection loop for the acquisition time, and passed to the ESI needle at 30 µl/min.

Sequential precursor (Pre) and neutral loss (NL) scans of the extracts produce a series of spectra revealing a set of lipid species containing a common head group fragment. Lipid species were detected with the following scans: PC and LysoPC, [M + H]^+^ ions in positive ion mode with Pre 184.1; PE and LysoPE, [M + H]^+^ ions in positive ion mode with NL 141.0; PA, [M + NH_4_]^+^ in positive ion mode with NL 115.0; PG, [M + NH_4_]^+^ in positive ion mode with NL 189.0 for PG; PI, [M + NH_4_]^+^ in positive ion mode with NL 277.0; PS, [M + H]^+^ in positive ion mode with NL 185.0; LysoPG, [M − H]^−^ in negative mode with Pre 152.9. The collision gas pressure was set at 2 (arbitrary units (au)). The collision energies, with nitrogen in the collision cell, were +40 V for PC, +28 V for PE, +25 V for PA, +22 V for PG, PI and PS, and −57 V for LysoPG. Declustering potentials were +100 V for PC, PE, PA, PG, PI, and PS, and −100 V for LysoPG. Entrance potentials were +14 V for PC, PA, PG, PI, and PS, +15 V for PE, and −10 V for LysoPG. Exit potentials were +14 V for PC, PA, PG, PI, and PS, +11 V for PE, and −14 V for LysoPG. The mass analyzers were adjusted to a resolution of 0.7 u full width at half height. For each spectrum, 9 to 150 continuum scans were averaged in multiple channel analyzer (MCA) mode. The source temperature (heated nebulizer) was 100°C, the interface heater was “on”, and +5.5 kV or −4.5 kV were applied to the electrospray capillary. The curtain gas was set at 20 au, and the two ion source gases were set at 45 au.

Processing of the data, including isotope deconvolution, was done similar to the way described by Singh et al. [25]. The background of each spectrum was subtracted, the data were smoothed, and peak areas were integrated using a custom script and Applied Biosystems Analyst software. The lipids in each class were quantified in comparison to the two internal standards of that class. The first and typically every 11^th^ set of mass spectra were acquired on the internal standard mixture only. Peaks corresponding to the target lipids in these spectra were identified and molar amounts were calculated in comparison to the internal standards on the same lipid class. To correct for chemical or instrumental noise in the samples, the molar amount of each lipid metabolite detected in the “internal standards only” spectra was subtracted from the molar amount of each metabolite calculated in each set of sample spectra. The data from each “internal standards only” set of spectra were used to correct the data from the following 10 samples. The analyzed data (in nmol) were normalized to the sample's “dry lipid weight” to produce data in the units nmol/mg dry lipid weight. Finally, the data were expressed as mole percent of total lipids analyzed.

#### Sphingolipid Quantification

The ESI-MS/MS procedure for SL quantification was similar to that for PGL quantification. Lipid species were detected with the following scans: CER, [M + H − H_2_O]^+^ ions in positive ion mode with Pre 300 and Pre 328; IPC, [M − H]^−^ ions in negative ion mode with Pre 259; MIPC, [M − H]- ions in negative ion mode with Pre 421; M(IP)2C, [M − H]^−^ in negative mode with Pre 663.1. The internal standard, 16∶0–18∶1-PI, was detected with [M − H]^−^ in negative ion mode with Pre 241.0, as described in the previous section. The CER internal standard, t18:0-phytoceramide, was detected with [M + H − H_2_O]^+^ in positive ion mode with Pre 300. The collision gas pressure was set at 2 au. The collision energies, with nitrogen in the collision cell, were +43 V for CER, −72 V for IPC, −80 V for MIPC, and −75 V for M(IP)_2_C. The declustering potential was +180 V for CER and −180 V for IPC, MIPC, and M(IP)_2_C. The exit potential was +10 V for CER and −15 V for IPC, MIPC, and M(IP)_2_C. The mass analyzers were adjusted to a resolution of 0.7 u full width at half height. For each spectrum, 125 to 250 continuum scans were averaged in multiple channel analyzer (MCA) mode. The source temperature (heated nebulizer) was 100°C, the interface heater was “on”, and +5.5 kV or −4.5 kV were applied to the electrospray capillary. The curtain gas was set at 20 au, and the two ion source gases were set at 45 au. Of note, by collision induced dissociation, IPC molecular ions fragment to produce mass spectral fragments unique to IPC's. Notably, head group fragment of m/z 259 might be produced from the secondary fragmentation of arachidonates and other ions. However, by using a combination of characteristic fragmentation pattern of IPC's and stringent data processing, we could focus specifically on IPC molecular ions. The rest of the processing was similar to that for phosphoglycerides; however, all SL signals were normalized to the signal for 0.23 nmol 16∶0–18∶0-PI that was added in as an internal standard.

SL amounts were determined by normalizing the mass spectral signal so that a signal of 1.0 represents a signal equal to the signal of 1 nmol 16∶0–18∶0-PI (the internal standard). These data were then divided by the sample dry weight to obtain signal/dry weight, by the total SL signal to obtain % of total SL signal. It is possible that there is variation in ionization efficiency among various SLs and between the internal standard and SL species. Thus, normalized SL species amounts may not reflect their molar amounts. However, the employed procedure allows for determination of relative abundance of SLs and comparison of amounts of particular SLs among samples.

#### Sterol esters, di- and tri-acyl glycerol quantification

The ESI-MS/MS procedure for SE, DAG and TAG quantification was similar to that of PGL quantification. Precise amounts of internal standards were added in the following quantities (with small variations of amounts in different batches of internal standards): 4.6 nmol di15:0-DAG, 3.1 nmol tri17:0-TAG. Lipid species for SE, DAG and TAG were detected with the following scans: 16∶1, 16∶0, 18∶3, 18∶2, 18∶1 and 18∶0 -containing species, were detected as [M + NH_4_]^+^ in positive ion mode with NL 271.2, 273.2, 295.2, 297.2, 299.2 and 301.2 respectively. The internal standard, tri17:1-TAG was detected with [M + NH_4_]^+^ in positive ion mode with NL 285.2, and di15:0-DAG was detected with [M + NH_4_]^+^ in positive ion mode with NL 259.2. The collision gas pressure was set at 2 au. The collision energy, with nitrogen in the collision cell, was +25 V for SE, DAG and TAG. The declustering potential was +100 V for SE, DAG and TAG. The exit potential was +12 V for SE, DAG and TAG. The mass analyzers were adjusted to a resolution of 0.7 u full width at half height. For each spectrum, 50 continuum scans were averaged in multiple channel analyzer (MCA) mode. The source temperature (heated nebulizer) was 100°C, the interface heater was “on”, and +5.5 kV were applied to the electrospray capillary. The curtain gas was set at 20 au, and the two ion source gases were set at 45 au. The rest of the processing was similar to that for PGLs; however, all SE signals were normalized to the signal for 4.6 nmol di15:0-DAG that was added in as an internal standard.

For these samples, all SE, DAG and TAG are represented as a list of “fatty acid containing” species. So, there are the 16∶0 containing species, the 18∶1 containing species and so on. There is no total DAG or TAG, as some of these species overlap with each other. For example, 16∶0–18∶1 DAG will appear in both the 16∶0 containing and the 18∶1 containing list. The data for DAG and TAG has been described as “Relative mass spectral signal”, where the unit is the signal for 1 nmol of the internal standard. The data are normalized to dry lipid weight. Thus, these amounts are not true molar amounts, but they can be used to compare the amounts of particular molecular species among samples.

SE amounts were determined by normalizing the mass spectral signal so that a signal of 1.0 represents a signal equal to the signal of 1 nmol di15:0-DAG (the internal standard). The SE data were then divided by the dry lipid weight to obtain signal/dry lipid weight, by the total SE signal to obtain % of total SE signal. It is possible that there is variation in ionization efficiency among various SEs and between the internal standard and SE species. Thus, normalized SE species amounts may not reflect their molar amounts. However, the employed procedure allows for determination of relative abundance of SEs and comparison of amounts of particular SEs among samples. Of note, the total amount of each SE class has been calculated by adding the normalized mass spectral signal of all respective FA containing SE (for example, total amount of ergosterol esters was calculated by adding the mass spectral signal of 16∶1, 16∶0, 18∶3, 18∶2, 18∶1 and 18∶0 –containing ergosterol ester).

### Statistical Analysis

The mean of three independent biological replicates ± standard deviation (SD) from the individual samples was used to compare the lipids of *Candida* species. Multivariate data analysis (pattern recognition) was employed. Principal component analysis (PCA) was performed using the software SYSTAT, version 10 (Systat Software Inc., Richmond, CA, USA) using three replicates of each of the AS and AR *Candida* isolate to highlight the statistically significant lipid differences. To assess the statistical significance of the difference in lipid datasets and PC scores, the Student *t*-test was performed using the significance level of 0.05. When all the values for a particular lipid species were zero in all samples, the data for that lipid species were removed from the analysis. The data in percentage were log-transformed and normalized to the same scale for PCA.

## Results

To analyze the global lipidomic changes associated with azole resistance, we used a large number of genetically matched pairs of AR isolates. All the AR isolates were similar, as they showed high MIC_80_ for fluconazole and other drugs; however, based on the mechanism of acquiring azole resistance, they could be segregated into two distinct groups (Supplementary Table S1). For example, while in the first group which included Gu4/Gu5, DSY294/DSY296 DSY347/DSY289 and DSY544/DSY775 pairs, azole resistance was predominantly attributed to an over-expression of an ABC transporter encoding gene CaCDR1 [27], [28], and for the second set of pairs G2/G5, F2/F5, DSY290/DSY292 and DSY741/DSY742, the resistance to azoles was mainly due to an over-expression of MFS encoding gene, *CaMDR1* [29], [30]. The availability of highly related AS and AR isolates enabled us to make a direct comparison of their lipidomes.

For lipidome analysis, AS and AR *Candida* cells were harvested in the exponential growth phase and their total lipids were extracted as described in Materials and Methods [23]. The extracted lipids were subjected to ESI-MS/MS by direct infusion of the lipid extracts. The total lipids (PGLs, SLs and SEs) were quantified and lipid content (as total normalized mass spectral signal of PGL + SL + SE) was found to range between to 453 to 1116 nmol per mg dry lipid weight of AS and AR isolates (Supplementary Sheet S1). A comparison of lipidome of AS and AR isolates did not give a typical pattern of variations, however, some of the differences appeared to be more prominent among the AR isolates. To highlight the changes between AS and AR isolates, we selected and discussed a few as an example. Although, we determined lipids to their absolute amounts (as total normalized mass spectral signal of PGL + SL + SE), we have used the mole percentages (as % of total normalized mass spectral signal of PGL + SL + SE) for data analysis, which have lower standard deviation. By employing MS analysis, nine major PGLs classes which included PC, PE, PI, PS, PG, PA LysoPC, LysoPE and LysoPG in AS and AR isolates were targeted. Additionally, lipid molecular species were identified by mass of the head group plus the mass of the intact lipid, allowing determination of the number of C atoms and double bonds in the acyl chain(s) of PGLs. The PGLs were quantified in relation to internal standards of the same lipid class. This procedure is known to provide accurate quantification because various molecular species of the same lipid class (here, the internal standard and other species) produce very similar amounts of mass spectral signal after electrospray ionization [31].

Our MS analysis also included four major groups of SLs, CER, IPC, MIPC, M(IP)_2_C and their relative amounts were determined. As discussed in Methods, SEs, DAG and TAG, were analyzed on the basis of their FA compositions (six major FAs were analyzed, including C16∶1, C16∶0, C18∶3, C18∶2, C18∶1 and C18∶0, as these are the most abundant FAs present in *Candida*) and their relative amounts were determined on the basis of respective internal standards.

### The PGLs and SLs compositional profile is different among various AS/AR matched pairs

**Figure 1:**
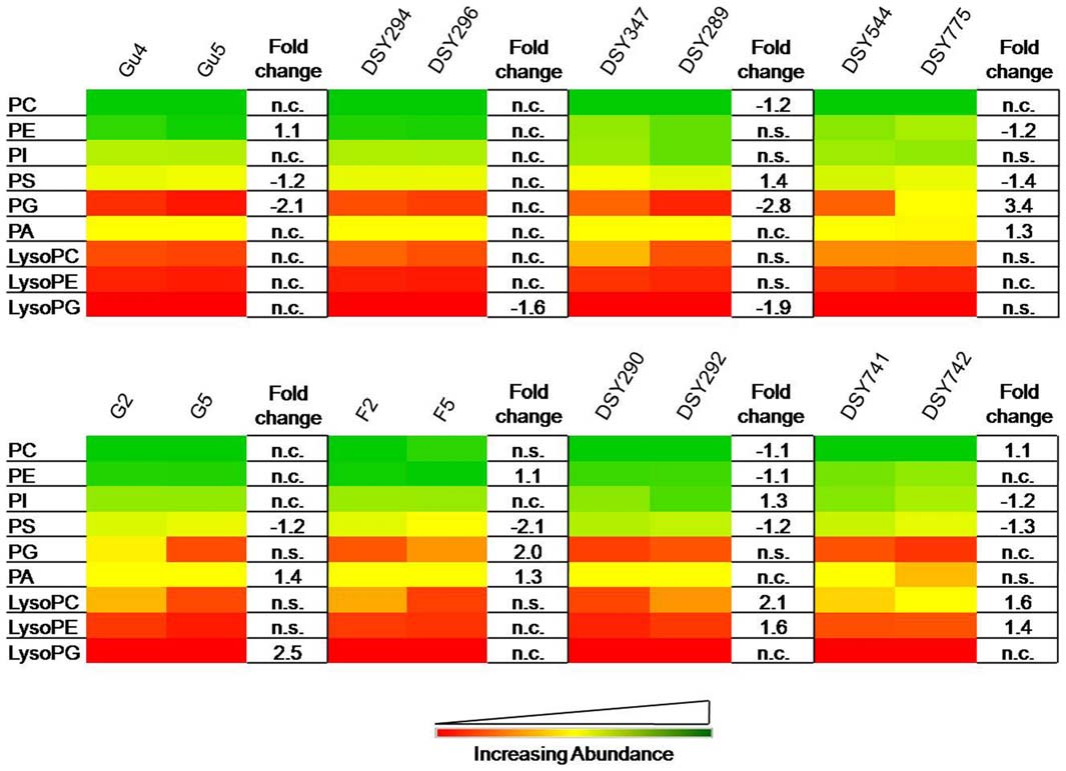
The composition of PGL classes in azole sensitive (AS) and resistant (AR) isolates of *C. albicans*. Data in the heat map is represented as % of the total PGL signal after normalization to internal standards. Values are means ± SD (n = 3–5 for all *Candida* strains). Statistically significant fold changes have been depicted in columns right to each heat map panel (P<0.05). No change is depicted by ‘n.c.’ and statistically insignificant change (P>0.05) is depicted by ‘n.s’. Green, yellow and red color depicts the highest, mid and lowest values respectively. Data taken from Supplementary Sheet S2.

Our method could detect PC, PE, PI, PS, PG and PA as major PGLs among AS and AR isolates. The abundance of PGLs was in order PC, PE, PI, PS, PA, PG, which did not change between the AS and AR pairs (Figure 1). PC, PE and PI accounted for almost 80% PGLs in all the isolates. As shown in Figure 1, fluctuations in PGL levels are quite evident among all AS/AR pairs except in the pair DSY294/DSY296, where no significant change was observed. The contents of PS decreased by as much as 2 fold among all AR isolates, except in DSY289 where it increased by 1.4 fold. However, in DSY296, there was no change in PS content. It is noteworthy that PS is significantly found to be lower in all those AR isolates where the MFS transporter CaMdr1p is overexpressed. Other lipids, namely PC, PE, PI, PG and PA showed minor but significant changes among various AS/AR pairs, but these changes were not consistent between pairs. Generally, among the three major lyso-PGLs analyzed, namely LysoPC, LysoPE and LysoPG, no significant differences were observed among the majority of AS/AR isolates. However, LysoPG content was up to 2.5 times lower in DSY296, DSY289 and in G5 isolates, while LysoPC and LysoPE content increased up to 2 folds in DSY292 and DSY742 isolates.

**Figure 2 pone-0019266-g002:**
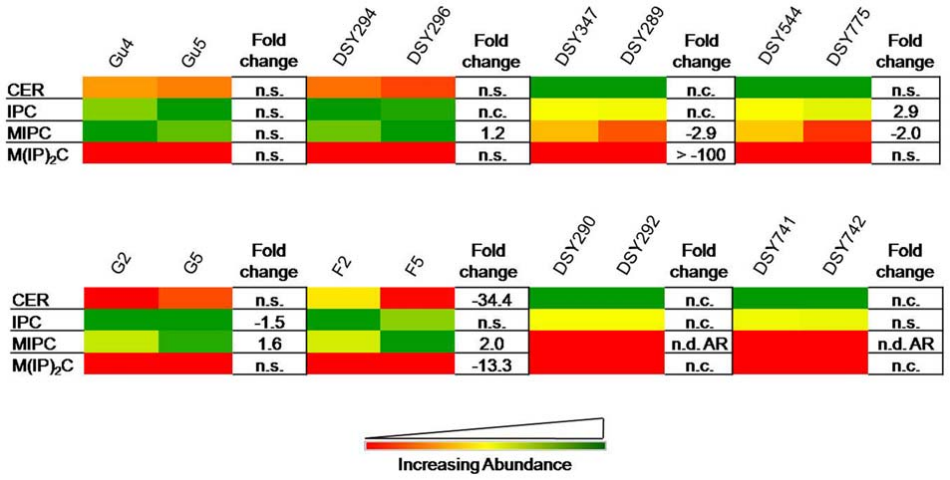
The composition of SL classes in AS and AR isolates of *C. albicans*. Data in the heat map is represented as % of the total SL signal after normalization to internal standards. Values are means ± SD (n = 3–5 for all *Candida* strains). Statistically significant fold changes have been depicted in columns right to each heat map panel (P<0.05). No change is depicted by ‘n.c.’ and statistically insignificant change (P>0.05) is depicted by ‘n.s’. ‘n.d.A.R.’ depicts that the particular lipid class was not detected in AR isolate. Green, yellow and red color depicts the highest, mid and lowest values respectively. Data taken from Supplementary Sheet S2.

Four major SL groups including CER, IPC, MIPC and M(IP)_2_C, were detected. While the M(IP)_2_C abundance, which is the most complex phospho-SL, was found to be <2.2% among all analyzed isolates, MIPC, was the most variable SL. It was depleted over 2 folds in DSY289, DSY775, DSY292 and DSY742 AR isolates while it was raised up to 2 folds in DSY296, G5 and F5 isolates (Figure 2).

### SE homeostasis is altered among various AS/AR matched pairs

**Figure 3:**
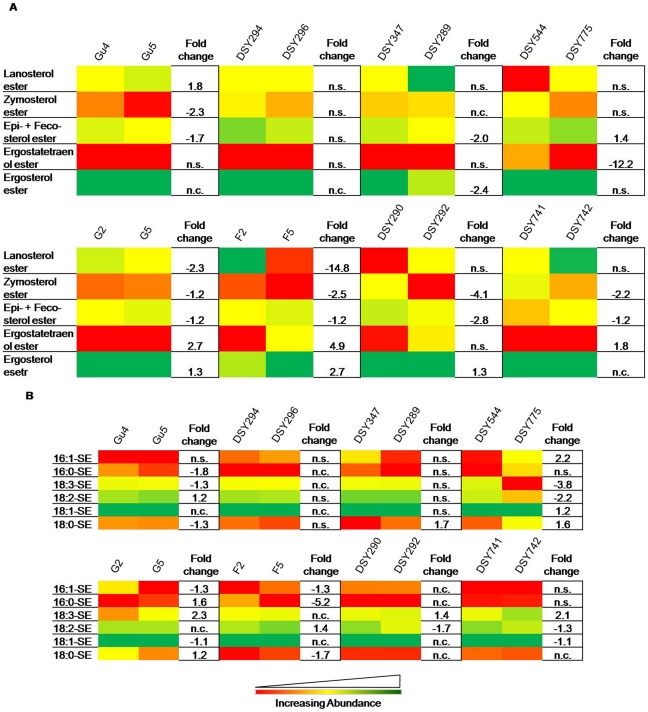
The composition of SE classes in AS and AR isolates of *C. albicans*. Data in the heat map is represented as % of the total SE signal after normalization to internal standards. A) SE composition based upon major FAs. B) SE composition of major sterols among AS/AR isolates. Values are means ± SD (n = 3–5 for all *Candida* strains). Statistically significant fold changes have been depicted (P<0.05). No change is depicted by ‘n.c.’ and statistically insignificant change (P>0.05) is depicted by ‘n.s’. Green, yellow and red color depicts the highest, mid and lowest values respectively. Data taken from Supplementary Sheet S2.

Sterols were identified and quantified as SE. Lanosterol, zymosterol, episterol, fecosterol, ergostatetraenol and ergosterol were the major components in all AS/AR pairs, which ranged between 1–80% of the total SEs (Figure 3A). The intermediate metabolites of sterol biosynthetic pathway such as epi- and feco- and zymo- esters were ∼1.2 to 4 fold depleted in Gu5, DSY289, G5, F5, DSY292, DSY742 AR isolates. Lanosterol esters which are also important sterol biosynthesis precursor were depleted 2–15 folds in F5 and G5 isolates. The ergostatetraenol and ergosterol esters, which are the end products of the sterol biosynthesis, were significantly up by 1.3–5 folds in G5, F5, DSY292 and DSY742 AR isolates (Figure 3 A). While examining the FA composition of these SEs, we found that 18∶3-SE specifically depleted in Gu5 and DSY775, and elevated in G5, DSY292 and DSY742. Notably, none of these SEs changed significantly in DSY294/DSY296 pair (Figure 3B).

### DAGs and TAGs show variations among various AS/AR matched pairs

DAGs were found to be significantly depleted (by ∼1.3–5 fold) among DSY 296, DSY289, DSY775 and G5 AR isolates, while they increased (by ∼1.4–10 fold) among F5, DSY292 and Gu5 AR isolates (Supplementary Figure S1). In DSY742, only 18∶3-DAG content was increased by 1.6 folds. Similarly, TAGs were found to be significantly depleted (by 1.4 fold or more) among Gu5, DSY289 and DSY775 AR isolates, and increased (by 1.5 fold or more) among G5, F5 and DSY742 AR isolates (Supplementary Figure S2). However, there was no change in TAG contents of DSY296 and DSY742 AR isolates (Supplementary Figure S2).

### Molecular lipid species show ripple effects on lipidome of AR isolates

**Figure 4:**
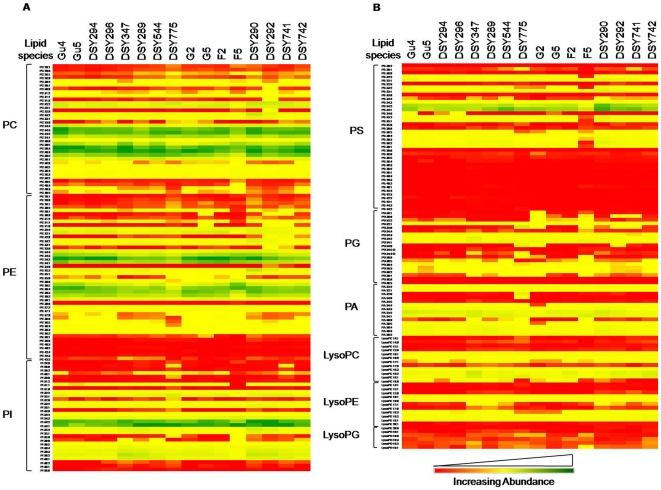
Molecular lipid species composition of AS and AR isolates. A) Molecular lipid species composition of PGLs PC, PE and PI (as % of PGL mass spectral signal normalized to the internal standards). B) Molecular lipid species composition of PGLs PS, PG, PA, LysoPC, LysoPE and LysoPG (as % of PGL mass spectral signal normalized to the internal standards). *Candida* strains were cultured in YPD medium at 30°C as described in Materials and Methods. Data in the heat map are means ± SD (n = 3–5, more than three independent analysis of lipid extracts from more than three independent cultures). Green, yellow and red color depicts the highest, mid and lowest values respectively. Data taken from Supplementary Sheet S2.

**Figure 5:**
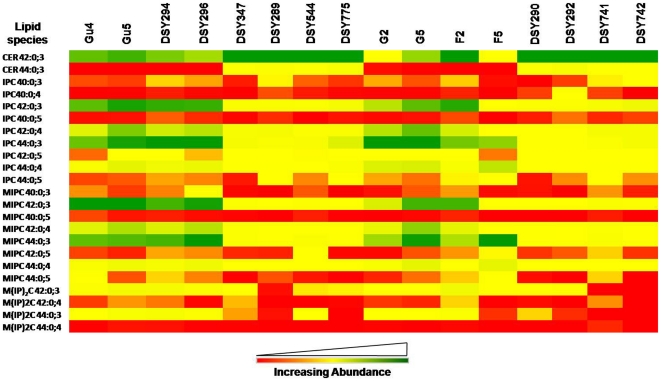
Molecular SL species composition of AS and AR isolates of *C. albicans*. Data in the heat map is represented as % of SL mass spectral signal normalized to the internal standards *Candida* strains were cultured in YPD medium at 30°C as described in Materials and Methods. Values are means ± SD (n = 3–5, for all *Candida* strains). Green, yellow and red color depicts the highest, mid and lowest values respectively. Data taken from Supplementary Sheet S2.

Each lipid class is sub-categorized into its molecular species which differs from others in fatty acid composition and their positional distribution [32]. By mass spectrometric analysis of the extracts of AS and AR pairs, we detected molecular lipid species belonging to five major lipid groups (PGL, SL, SE, DAG and TAG; see Supplementary Sheet S2). We could determine the abundance of over 260 species belonging to PGL, SL and SEs (Figure 3A, 4, and 5). However, for DAGs and TAGs the data is rather relative, but nonetheless can be used for comparisons between AS/AR isolates (as described in methods). The total number of species detected for each of the AS/AR pairs was mostly the same (Supplementary Sheet S1). For example, over 200 PGL species were quantitatively detectable among all AR isolates. However, considerable difference existed in terms of relative abundance of different lipid species between AS and AR isolates. For example, out of 242 lipid species of 18 major lipid classes (PGL + SL + SE), among *CaCDR1* attributed pairs, only the pair DSY544/DSY775 showed significant differences in ∼128 lipid species, while other pairs did not show much variation in lipid species (∼18–70 species) (Supplementary Sheet S3). In contrast, those pairs where azole resistance was due to *CaMDR1* over-expression, ∼66–127 lipid species were significantly different (Supplementary Sheet S3). Evidently, the lipidome reshuffling ranges from minimal to huge in terms of molecular lipid species among AS/AR pairs. Based upon these differences we sorted out a list of lipid species that show modest to robust variations among the AR isolates (Supplementary Sheet S3). These generally include mono- to poly- unsaturated PC, PE, PG, PA and PI species containing 36 to 40 carbons, lyso-PGLs (LysoPC 16∶0, LysoPE 18∶2 and LysoPE 18∶3), MIPC 56∶0;3 and zymosterol ester.

### Molecular lipid species differences are validated by the principal component analysis

To confirm if the observed changes in molecular species of lipids represent statistically significant variations between AS and AR isolates, the principal component analysis (PCA) was performed. PCA is a mathematical algorithm that reduces the dimensionality of the data while retaining most of the variation in the data set [33]. The data are represented by principal components, with the first principal component accounting for the maximum possible variation in the data, and each succeeding component accounting for a portion of the remaining possible variation. Plots allow visual assessment of the similarities and differences among samples and help to determine whether and how samples can be grouped [24], [25], [34]. We performed PCA using the molecular species percentage composition of PGL + SL + SE (data in Supplementary Sheet S1) to identify and highlight the AR-specific, statistically significant lipid differences.

**Figure 6:**
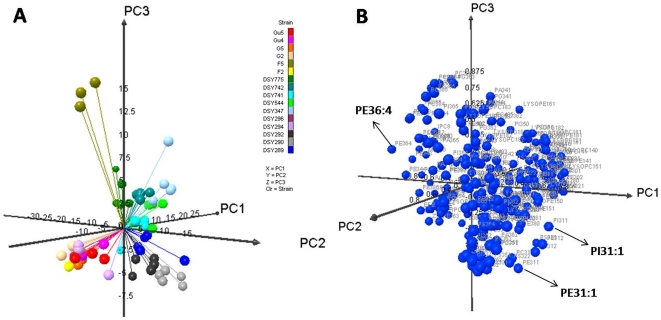
Principal component analysis (PCA) of lipid species amongst all AS and AR isolates of *C. albicans*. The figure shows the 3D- PCA (Figure 6 A) and loading (Figure 6B) score plots for all AS/AR isolates. The scores for the first three principal components, explaining >40% of the variance, are plotted. Each point in the PCA plot represents the principal component score of the individual replicate. Based upon the loading scores, few examples of molecular lipid species that are most variable among the AS/AR pairs have been marked with arrows. Data taken from Supplementary Sheet S4.

To identify the lipid species changes, PCA was first done on combined data sets of all isolates. The 3-D PCA plot on all combined AS and AR datasets confirmed that all AS/AR pairs have unique lipid profiles as they did not segregate as different groups of isolates (Figure 6 and Supplementary Sheet S4). The highest and lowest loading values reflect the lipid species that are most important variable in the assignment of each principal component (Supplementary Sheet S4). For example, examination of the loadings associated with principal component 1 (Supplementary Sheet S4) shows that the molecular species contents of IPCs and MIPCs are important for the separation of AS/AR *Candida* along with the negative principal component 1 axis, while predominantly mono- or di- unsaturated carbon PGLs namely PC's (26∶1, 28∶1 and 31∶2), PE 30∶2, PA's (32∶1 and 32∶2) and PI 31∶1 are important determinants for the separation of AS/AR isolates along the positive axis. On the other hand, principal component 2 differentiates several AS/AR *Candida* with saturated or mono-unsaturated lipid species (PE (30∶1, 32∶1, 37∶0), PI (30∶0, 30∶1, 32∶1), PG (28∶0, 31∶0, 32∶0, 34∶0), PC 30∶1 and PI 32∶1) along with the negative axis while with poly-unsaturated long chain lipid species (PS (34∶4, 36∶3, 36∶5), PE (42∶2, PA 36∶5) and PI (36∶4, 36∶5) along with the positive axis. Similarly, the principal component 3 is defined by the amino-PGLs, PE and PS species, along with the negative axis, while PG and PC species along with the positive axis. Our PCA analysis of all the isolates did reveal significant variations in the molecular lipid species of AS/AR isolates. Notably, PCA analysis done on the dataset of all AS isolates together and all AR isolates together, as two groups, highlighted the variation in molecular species amongst the isolates of the respective group; but could not highlight the common variables between the AS/AR pairs (Supplementary Figure S3, S4 and Sheet S4).

### Several common molecular lipid species are more susceptible to variation in response to azole

To focus on the molecular lipid species that could commonly vary between each AS and AR pair, PCA analysis was done on the individual pairs (Supplementary Figure S5A). The loading scores associated with the principal component 1, 2 and 3 are summarized in the Supplementary Sheet S4 (Supplementary Figure S5B). Upon careful examination of the loading values of the principal component 1, we could identify 17 different molecular PL species, whose composition consistently varied in most of the AR isolates (Supplementary Table S2). These included species ranging from mono-unsaturated lipid species (for example, PC30∶1, PC32∶1, PI30∶1, PI34∶1) to poly-unsaturated lipid species (PC36∶4, PC36∶5, PE36∶4, PE36∶5, etc.). Consistent variation among some MIPC (MIPC 42∶0;3) and SE species was also evident (Supplementary Table S2). Supplementary Figure S6 depicts four typical variable molecular lipid species of PGLs between AS and AR isolates.

## Discussion

**Figure 7:**
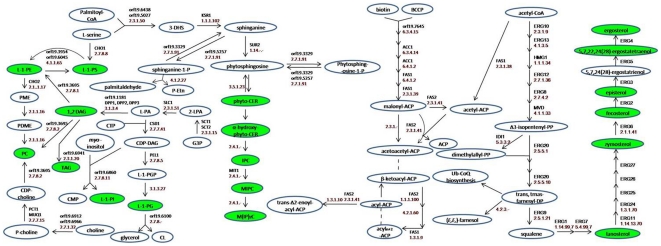
Partial lipid metabolic pathway of *Candida*. The lipid metabolic network was compiled using *Candida* genome database (www.candidagenome.org) and references therein. Enzymes are annotated by gene name. The green color depicts the lipid classes that were identified and quantified for AS/AR isolates.

Although the importance of lipids in the physiology of *Candida* including in MDR has been realized [9]–[15], this represents the first study to dissect and evaluate lipid metabolome of AS and AR isolates. By using a high throughput mass spectrometry based lipidome analyses of pathogenic *C. albicans*, we have performed comparative lipidomics between AS and AR matched pair isolates and could determine the abundance of 16 major lipid classes, which provided a significant coverage of the lipid metabolic network in *Candida* (Figure 7). We observed that the relative abundance of major classes of PLs remained similar between AS and AR pairs. However, the lipid profile of AR isolate of each pair was typical (Figure 1–3, Supplementary Figure S1 and S2). What emerges from this comparative analysis is that there is no typical lipid composition which could be directly linked to azole resistance. Considering that membrane lipids are the target of environmental stresses and that each pair of AS and AR *Candida* has been isolated from different host, typical metabolic state of each pair is not surprising. This is also supported by the fact that the lipidome of AS isolates themselves differ from each other. Thus each AS *Candida* strain appears to have a mechanism to sense and respond to environment and change its lipid composition. The differences in lipid composition due to azole stress necessitate compensatory changes in other lipids. For example, PS is depleted among most AR isolates; however, the contents of PE which is formed after decarboxylation of PS are largely maintained (Figure 1). This supports the fact that defects in *de novo* PE synthesis compromises virulence of *C. albicans* [35]. PG is other PGLs which appeared to be more responsive to azole resistance as it was variable in all the isolates. It is noteworthy that PGL has earlier been implicated in optimal mitochondrial functions and maintenance of yeast susceptibility to azole antifungals [36].

While relative abundance of several major classes of lipids did not significantly vary between AS and AR isolates, a closer look revealed interesting insights at the molecular species level (Figure 4A and 4B). Of note, the molecular species of PGLs were only slightly different in terms of numbers identified in each pair, but varied considerably in mole percentages distribution (Figure 4A and 4B). The molecular lipid species differ from each other on the basis of FA-chain length, number of double bonds and their positional distribution, which play an important role in different cellular functions, by providing an excellent platform to the cell to remodel its metabolite level and maintain lipid homeostasis [24], [32]. We analyzed commonality between molecular lipid species which could help AR cells to maintain its lipid homeostasis. The data indicates that azole resistant *Candida* undergoes massive lipidome remodeling where certain common lipid species are more significantly changed between most of the AS and AR isolates (Figure 4 and Figure 5, Supplementary Table S2). These generally included mono- to poly- unsaturated PGL species containing 36 to 40 carbons and a few of SL and SE species (Figure 4 and Figure 5). It is thus apparent that azole resistant *Candida* shows a great deal of adaptability at the level of molecular lipid species to maintain its optimal membrane lipid environment. PCA analysis between individual AS and AR pair confirmed that the overall changes in the lipid profile, though typical between matched isolates, are all related with some commonalities at the molecular lipid species level (Supplementary Table S2). These common variable molecular species (for example, PC30∶1, PC32∶1, PI30∶1, PI34∶1, PC36∶4, PC36∶5, PE36∶4, PE36∶5, MIPC 42∶0;3, SE) among AS and AR isolates could be considered as more responsive to the drug stress.

Our pool of AR isolates included four pairs of each with either over-expressed *CaCDR1* or *CaMDR1* as major factor of azole resistance (Supplementary Table S1) [26]–[29]. The two proteins encoded by these genes, though functionally identical in term of drug extrusion, differ mechanistically in achieving it [5]. Another difference is that the two efflux pump proteins e.g. CaCdr1p and CaMdr1p show discrete preference to their recruitment to plasma membrane [9]. For example, it has been shown that ABC transporter CaCdr1p is predominantly localized within sphingolipid and ergosterol rich micro-domains as compared to MFS transporter CaMdr1p which shows no such preference [9]. Based on this back ground, we examined our lipidome data set to assess if overexpression of the two proteins of different lipid preferences could be correlated with the changes in lipid homeostatic environment. Our data confirmed that indeed the overexpression of either of the two transport proteins in AR isolates elicited different lipidomic response. For example, among PGLs, PS amounts were consistently declined in all CaMdr1p overexpressing AR isolates (Figure 1). In contrast, ergostatetraenol and ergosterol esters were significantly accumulated among CaMdr1p overexpressing AR isolates (Figure 3A). None of the above changes were consistent in CaCdr1p over-expressing strains which typically showed depletion of signaling and storage neutral lipids like DAGs and TAGs levels (Supplementary Figure S1 and S2). It is well known that protein kinase C (PKC) is activated by the DAGs in the presence of PS as a cofactor [37]–[42]. Therefore, it is possible that these neutral lipids, along with PLs, might contribute to MDR development through very tightly regulated signaling cascades. Interestingly, PKC signaling pathway has been reported to contribute to antifungal tolerance in *C. albicans* and *S. cereviseae* [43], [44]. Some efflux protein dependent interesting patterns among molecular lipid species could also be observed. For example, while the contents of 18∶3-SE were specifically depleted in CaCdr1p overexpressing pairs, they were higher in CaMdr1p over-expressing pairs (Figure 3A). Furthermore, molecular lipid species ratios for PC and PE, 36∶6/36∶4 and 36∶6/34∶4 (18∶3-FA indicator), showed a decreasing trend among some CaCdr1p overexpressing AR isolates, while opposite was true among the CaMdr1p attributed AR isolates (Supplementary Figure S7). This comparison revealed that an overexpression of either of the two efflux proteins might necessitate typical changes in lipid profiles. It is also apparent that an overexpression of MFS transporter CaMdr1p is accompanied by more pronounced changes in lipid profiling as compared to CaCdr1p cells. This would imply that in addition to responding to azole stress, the lipidome of AR isolates might be also susceptible to the over-expression of membrane efflux pump proteins. This is not unexpected since differences in membrane environment can have a significant effect on the insertion, folding and functioning of membrane proteins [45]–[48]. However, the fact that these lipid profile changes could be due common regulation of MDR pump encoding genes and lipid metabolism cannot be ignored [49], [50]. For example, mammalian presynaptic serotonin transporter (SERT) is functionally overexpressed only in the presence of cholesterol [51], while lactose permease (LacY) in *E. coli* requires PE for proper folding [52], [53]. Thus, lipidomic differences in response to overexpression of either ABC or MFS transporter in AR isolates will affect the physical state of the membrane, which in turn could influence drug transport and substrate translocation. This aspect merits consideration in the overall scenario of MDR.

### Conclusion

Taken together, in this study using a combination of high throughput mass spectrometry and statistical validation methods, we provide the first evidence of metabolic reprogramming between AS/AR matched pair isolates. Most metabolic changes are evident at the molecular lipid species level between each AS/AR isolates. Our study also highlights general commonality among molecular lipid species in most AR isolates and lipid perturbations that might be directly or indirectly associated with the overexpression of either CaCdr1p or CaMdr1p. Notwithstanding the fact that our comparative lipidomics of AS and AR do not provide the direct metabolic state of these isolates as they would exist in the host environment, it does provide a snap shot of the lipidomic status of AR *Candida*. The molecular characterization of lipidome of AS and AR *Candida* isolates would serve as a starting resource point to link clinical/functional genomics with pathway specific signaling and gene/protein/metabolite expression and function in relation to multidrug resistance of pathogenic *Candida*. Our study also provides evidence that each AR isolate is rather unique in terms of its lipidome which reflects interplay of several genetic and host factors and could be an important consideration in designing therapeutic strategies.

## Supporting Information

Figure S1 **The composition of DAG classes among various AS and AR isolates of** ***C. albicans*** **.** Data in the heat map is represented as mass spectral signal per mg dry lipid wt. normalized to internal standards). Values are means ± SD (n = 3–5 for all *Candida* strains). Statistically significant fold changes have been depicted (P<0.05). No change is depicted by ‘n.c.’ and statistically insignificant change (P>0.05) is depicted by ‘n.s’. Green, yellow and red color depicts the highest, mid and lowest values respectively. Data taken from Supplementary Sheet S2.
(TIF)

Figure S2 **The composition of TAG classes among various AS and AR isolates of** ***C. albicans*** **.** Data in the heat map is represented as mass spectral signal per mg dry lipid wt., normalized to internal standards. Values are means ± SD (n = 3–5 for all *Candida* strains). Statistically significant fold changes have been depicted (P<0.05). No change is depicted by ‘n.c.’ and statistically insignificant change (P>0.05) is depicted by ‘n.s’. Green, yellow and red color depicts the highest, mid and lowest values respectively. Data taken from Supplementary Sheet S2.
(TIF)

Figure S3 **Principal component analysis (PCA) of lipid species amongst all AS isolates of** ***C. albicans*** **.** The figure shows the 3D- PCA (Figure 6 A) and loading (Figure 6B) score plots for all AS isolates. The scores for the first three principal components, explaining >40% of the variance, are plotted. Each point in the PCA plot represents the principal component score of the individual replicate. Some examples of relevant species changes have been marked out separately in loading plot.
(TIF)

Figure S4 **Principal component analysis (PCA) of lipid species amongst all AR isolates of** ***C. albicans*** **.** The figure shows the 3D- PCA (Figure 6 A) and loading (Figure 6B) score plots for all AR isolates. The scores for the first three principal components, explaining >40% of the variance, are plotted. Each point in the PCA plot represents the principal component score of the individual replicate. Some examples of relevant species changes have been marked out separately in loading plot.
(TIF)

Figure S5 **Pair-wise principal component analysis (PCA) of lipid species amongst all AS and AR isolates of** ***C. albicans*** **.** The figure shows the 3D- PCA (Figure 7A) and loading (Figure 7B) score plots for various AS/AR isolates. The scores for the first three principal components, >40% of the variance, were plotted. Each point in the PCA plot represents the principal component score of the individual replicate. Based upon the loading scores, few examples of molecular lipid species that are most variable between each AS/AR pair have been marked with arrows. Data taken from Supplementary Sheet S4.
(TIF)

Figure S6 **Examples of molecular lipid species which vary in commonality among AR isolates.** Box plots diagrams of cellular concentrations of PC30∶1, PE35∶2, PI34∶1, and PS33∶2 among various AS/AR isolates. Values are means ± SD (n = 3–5, for all *Candida* strains) determined by ESI-MS/MS.
(TIF)

Figure S7 **Pattern of lipid ratios of molecular species among various AS and AR isolates.** The figure depicts PC ratios 36∶6/36∶4 and 36∶6/34∶4, and PE ratios 36∶6/36∶4 and 36∶6/34∶4. The mol% composition was used to calculate the ratios (n = 3–5 for all *Candida* strains).
(TIF)

Sheet S1 **This sheet has the absolute amounts (nmol per mg dry lipid wt.) and the mol percentages of the molecular lipid.**
(XLS)

Sheet S2 **This sheet has the mole percentages of the molecular lipid species for all lipid groups except DAGs and TAGs which are represented in terms of their absolute relative abundance.**
(XLS)

Sheet S3 **This sheet shows the p-values of the different molecular lipid species among various azole sensitive (AS) and azole resistant (AR) pairs.**
(XLS)

Sheet S4 **This sheet shows the loading scores for the various Prinicipal Component Analysis (PCA) done.**
(XLS)

Table S1 **Properties of various strains used in the study.**
(DOC)

Table S2 **Most common molecular lipid species amongst AS/AR isolates as determined by PCA analyses of individual matched pair isolates.** Values are represented as fold change determined as [% amount of molecular species in AR/% amount of molecular species in AS].
(DOC)
