# Supplementary figures and images for: Comparative Lipidomics of Azole Sensitive and Resistant Clinical Isolates of Candida albicans Reveals Unexpected Diversity in Molecular Lipid Imprints
Source: PLoS One. 2011 Apr 29;6(4):e19266. doi: 10.1371/journal.pone.0019266 (PMC3084813; doi:10.1371/journal.pone.0019266)

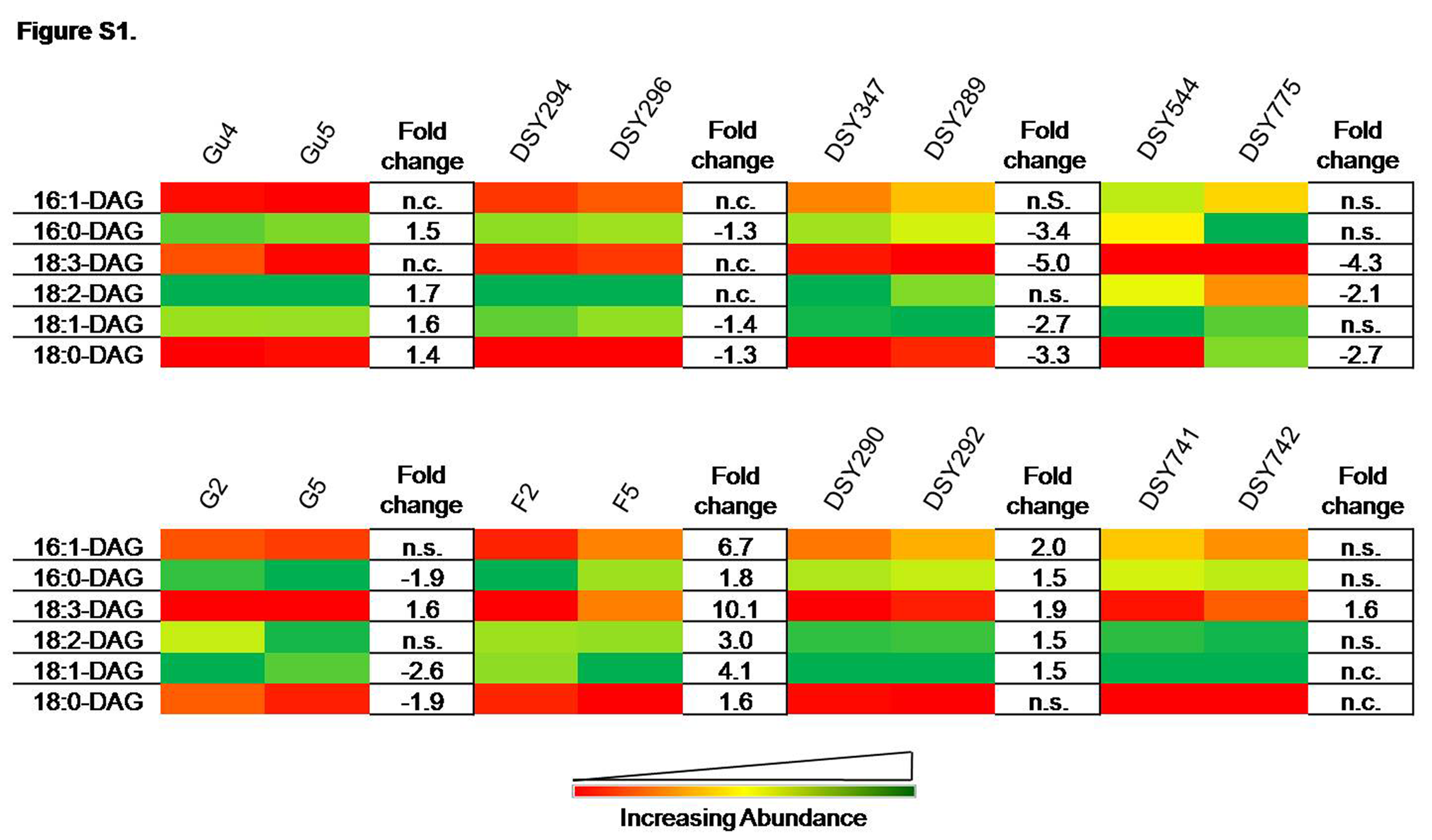

Supplement: Figure S1 — The composition of DAG classes among various AS and AR isolates of C. albicans . Data in the heat map is represented as mass spectral signal per mg dry lipid wt. normalized to internal standards). Values are means ± SD (n = 3–5 for all Candida strains). Statistically significant fold changes have been depicted (P<0.05). No change is depicted by ‘n.c.’ and statistically insignificant change (P>0.05) is depicted by ‘n.s’. Green, yellow and red color depicts the highest, mid and lowest values respectively. Data taken from Supplementary Sheet S2. (TIF) [file pone.0019266.s001.tif]

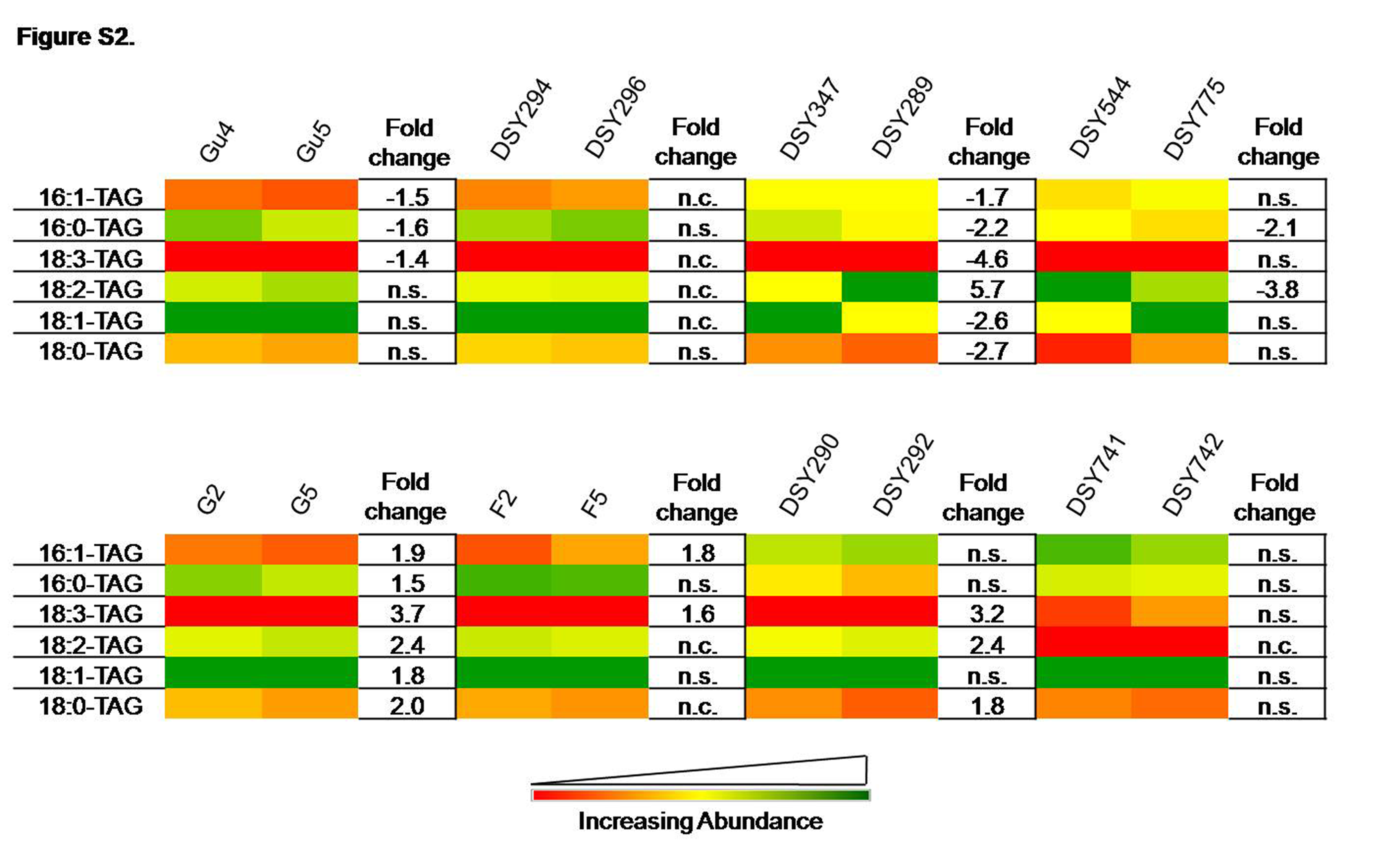

Supplement: Figure S2 — The composition of TAG classes among various AS and AR isolates of C. albicans . Data in the heat map is represented as mass spectral signal per mg dry lipid wt., normalized to internal standards. Values are means ± SD (n = 3–5 for all Candida strains). Statistically significant fold changes have been depicted (P<0.05). No change is depicted by ‘n.c.’ and statistically insignificant change (P>0.05) is depicted by ‘n.s’. Green, yellow and red color depicts the highest, mid and lowest values respectively. Data taken from Supplementary Sheet S2. (TIF) [file pone.0019266.s002.tif]

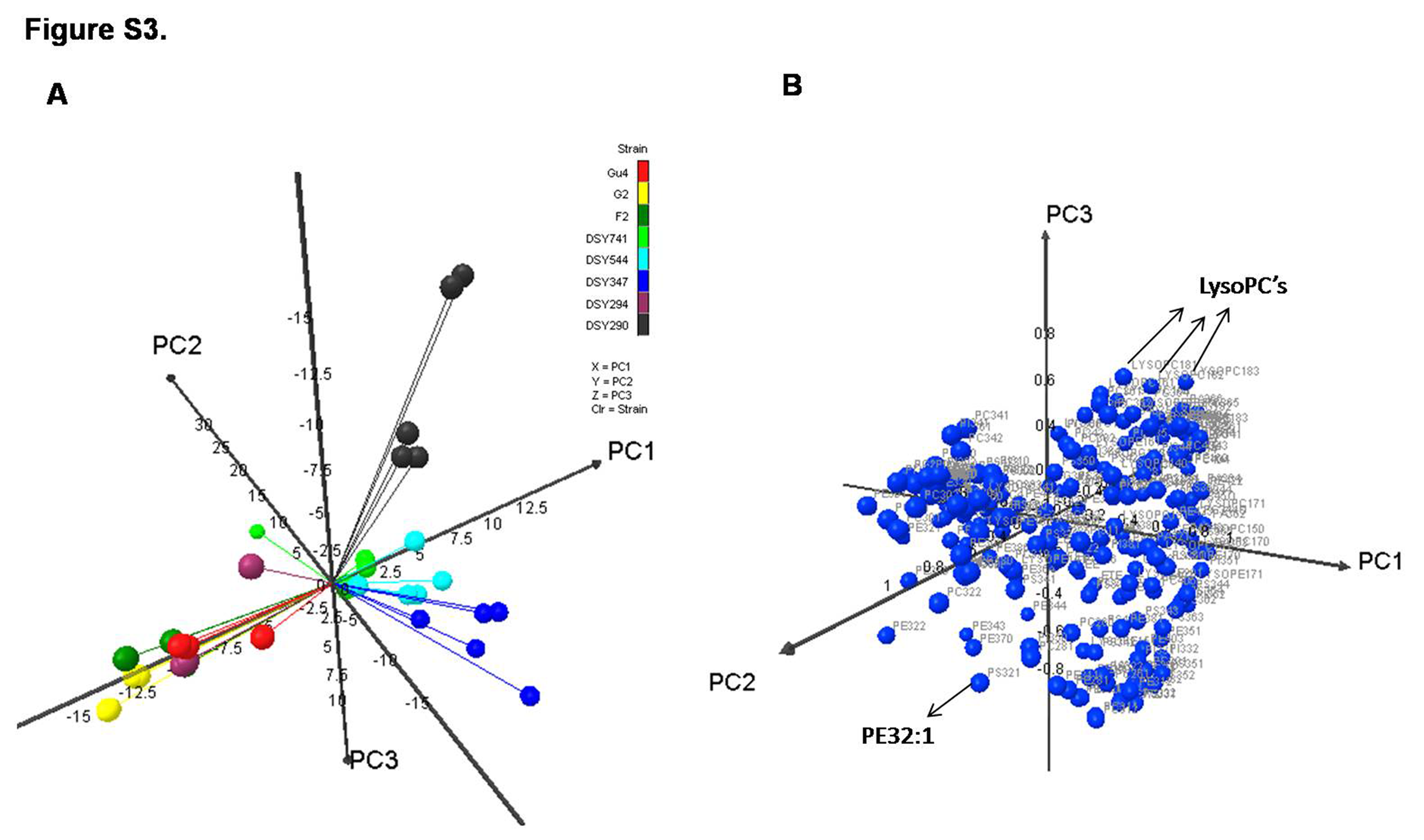

Supplement: Figure S3 — Principal component analysis (PCA) of lipid species amongst all AS isolates of C. albicans . The figure shows the 3D- PCA (Figure 6 A) and loading (Figure 6B) score plots for all AS isolates. The scores for the first three principal components, explaining >40% of the variance, are plotted. Each point in the PCA plot represents the principal component score of the individual replicate. Some examples of relevant species changes have been marked out separately in loading plot. (TIF) [file pone.0019266.s003.tif]

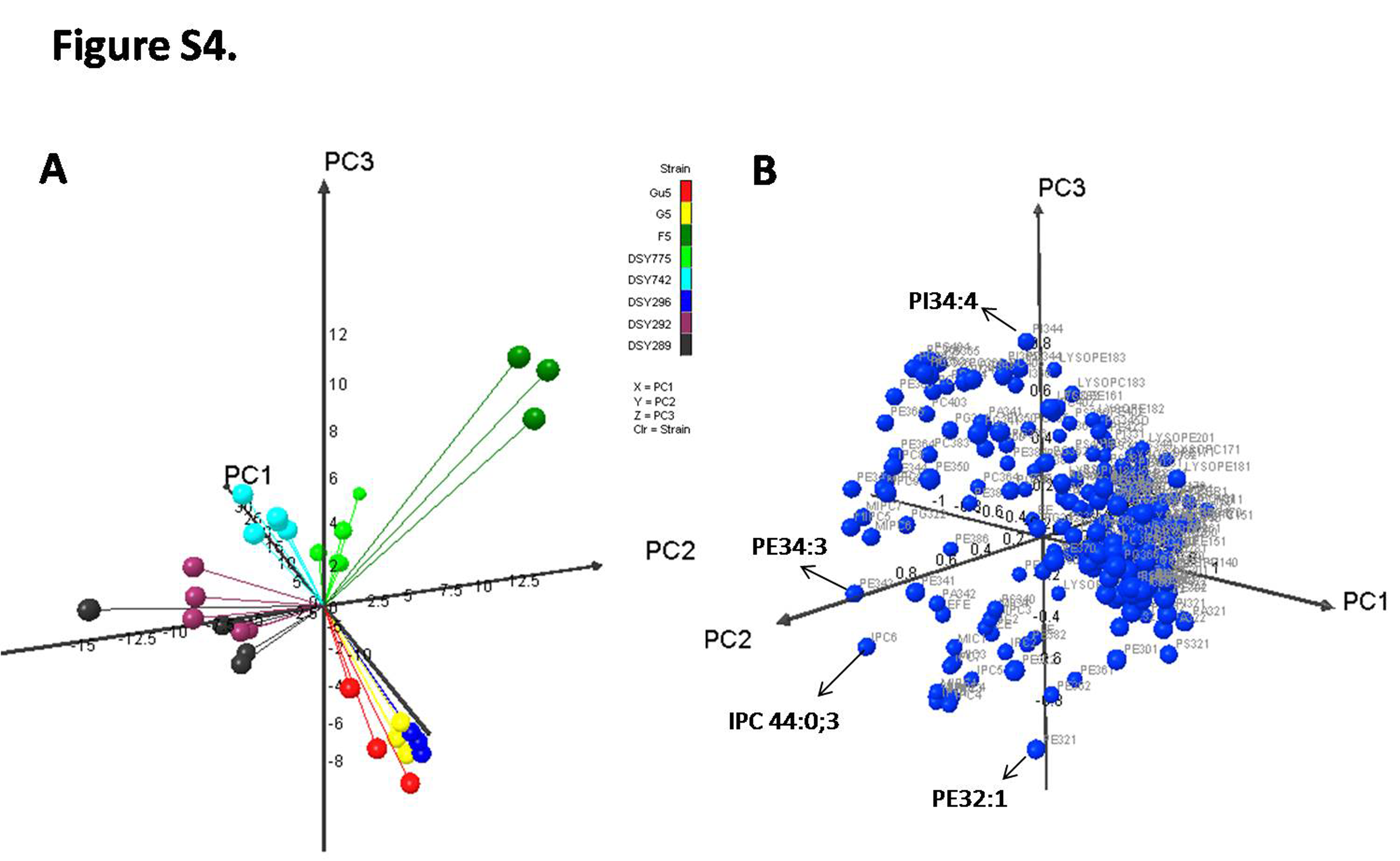

Supplement: Figure S4 — Principal component analysis (PCA) of lipid species amongst all AR isolates of C. albicans . The figure shows the 3D- PCA (Figure 6 A) and loading (Figure 6B) score plots for all AR isolates. The scores for the first three principal components, explaining >40% of the variance, are plotted. Each point in the PCA plot represents the principal component score of the individual replicate. Some examples of relevant species changes have been marked out separately in loading plot. (TIF) [file pone.0019266.s004.tif]

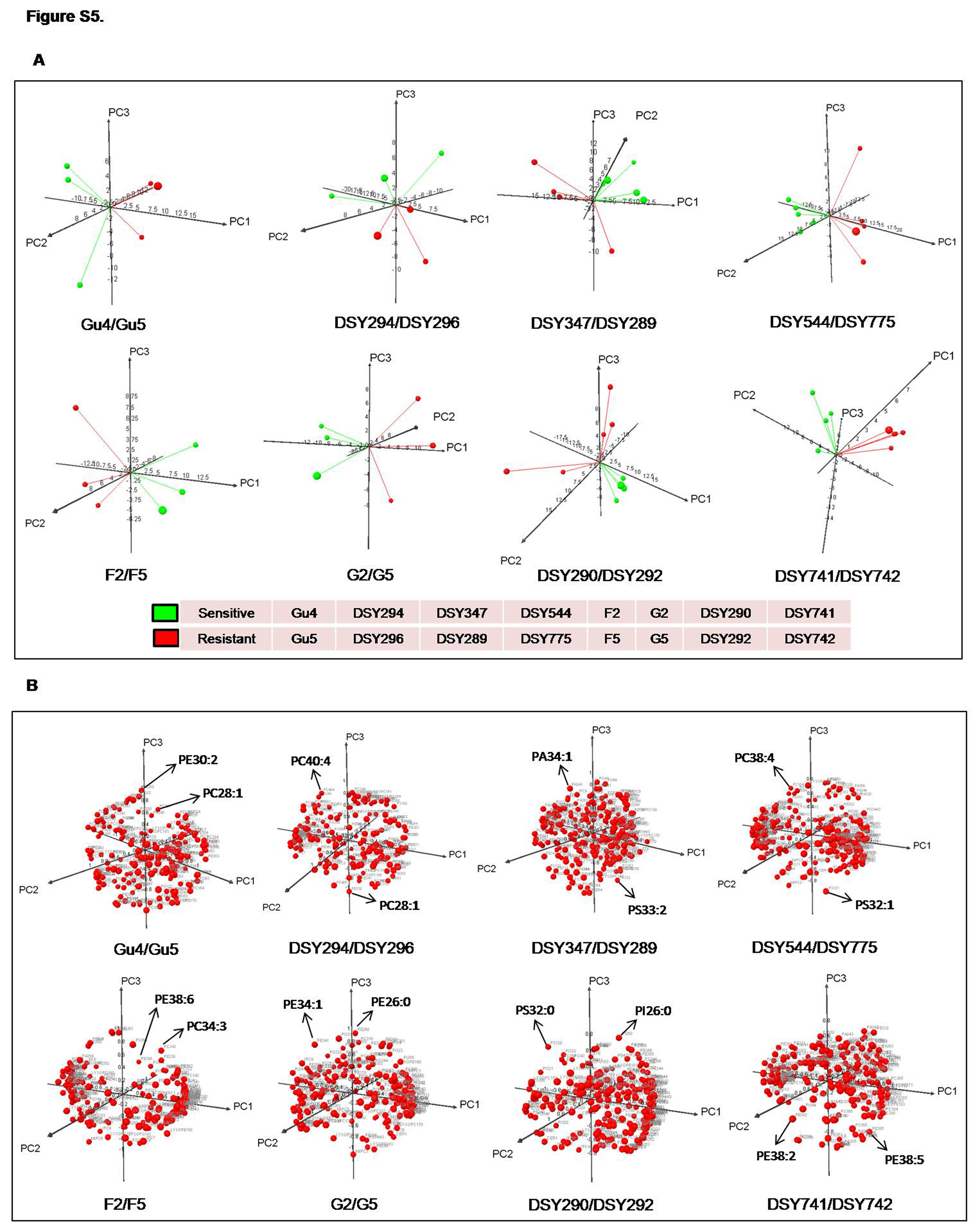

Supplement: Figure S5 — Pair-wise principal component analysis (PCA) of lipid species amongst all AS and AR isolates of C. albicans . The figure shows the 3D- PCA (Figure 7A) and loading (Figure 7B) score plots for various AS/AR isolates. The scores for the first three principal components, >40% of the variance, were plotted. Each point in the PCA plot represents the principal component score of the individual replicate. Based upon the loading scores, few examples of molecular lipid species that are most variable between each AS/AR pair have been marked with arrows. Data taken from Supplementary Sheet S4. (TIF) [file pone.0019266.s005.tif]

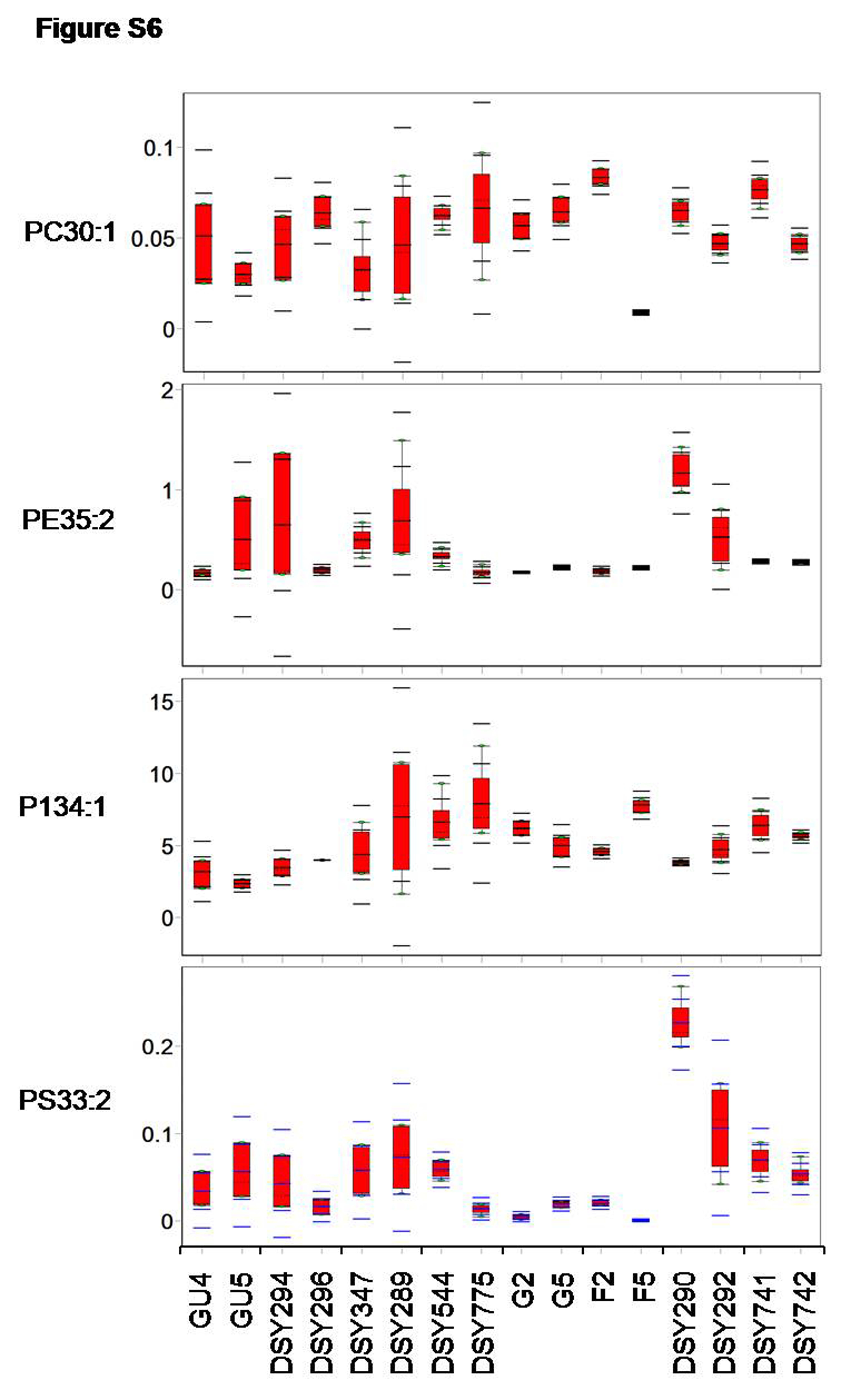

Supplement: Figure S6 — Examples of molecular lipid species which vary in commonality among AR isolates. Box plots diagrams of cellular concentrations of PC30∶1, PE35∶2, PI34∶1, and PS33∶2 among various AS/AR isolates. Values are means ± SD (n = 3–5, for all Candida strains) determined by ESI-MS/MS. (TIF) [file pone.0019266.s006.tif]

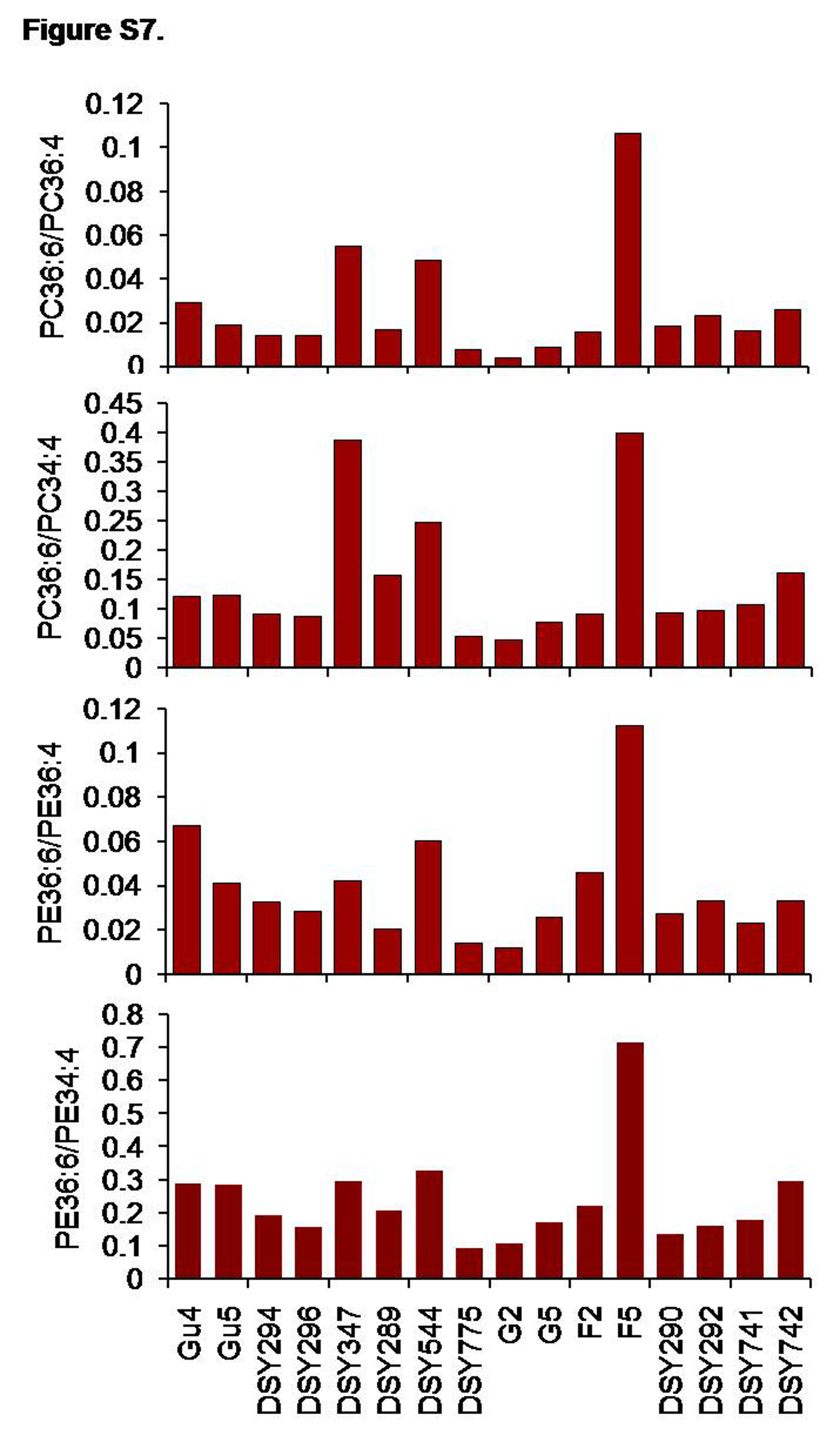

Supplement: Figure S7 — Pattern of lipid ratios of molecular species among various AS and AR isolates. The figure depicts PC ratios 36∶6/36∶4 and 36∶6/34∶4, and PE ratios 36∶6/36∶4 and 36∶6/34∶4. The mol% composition was used to calculate the ratios (n = 3–5 for all Candida strains). (TIF) [file pone.0019266.s007.tif]
