# Supplementary material for: Comparative Lipidomics of Azole Sensitive and Resistant Clinical Isolates of Candida albicans Reveals Unexpected Diversity in Molecular Lipid Imprints
Source: PLoS One. 2011 Apr 29;6(4):e19266. doi: 10.1371/journal.pone.0019266 (PMC3084813; doi:10.1371/journal.pone.0019266)
Supplement: Table S1 — Properties of various strains used in the study. (DOC) [file pone.0019266.s012.doc]

**Table S1. Properties of various strains used in the study.**

| **Strain** | **MIC_80_**  **(Fluconazole)** | ***CDR1/ CDR2*** | ***MDR1*** | **Reference** |
| --- | --- | --- | --- | --- |
| **Gu4** | 3.12 |  |  | [26] |
| **Gu5** | >100 | X |  | [26] |
| **DSY294** | 0.25 |  |  | [27] |
| **DSY296** | 128 | X |  | [27] |
| **DSY544** | 0.5 |  |  | This study |
| **DSY775** | 128 | X |  | This study |
| **DSY347** | 0.25 |  |  | This study |
| **DSY289** | 128 | X |  | This study |
| **G2** | 0.39 |  |  | [28] |
| **G5** | ≥50 |  | X | [28] |
| **F2** | 6.25 |  |  | [28] |
| **F5** | ≥50 |  | X | [28] |
| **DSY290** | 0.5 |  |  | This study |
| **DSY292** | 128 |  | X | This study |
| **DSY741** | 0.25 |  |  | This study |
| **DSY742** | 16 |  | X | This study |
