# Supplementary material for: Comparative Lipidomics of Azole Sensitive and Resistant Clinical Isolates of Candida albicans Reveals Unexpected Diversity in Molecular Lipid Imprints
Source: PLoS One. 2011 Apr 29;6(4):e19266. doi: 10.1371/journal.pone.0019266 (PMC3084813; doi:10.1371/journal.pone.0019266)
Supplement: Table S2 — Most common molecular lipid species amongst AS/AR isolates as determined by PCA analyses of individual matched pair isolates. Values are represented as fold change determined as [% amount of molecular species in AR/% amount of molecular species in AS]. (DOC) [file pone.0019266.s013.doc]

**Table S2. Most common molecular lipid species amongst AS/AR isolates as determined by PCA analyses of individual matched pair isolates. Values are represented as fold change determined as [% amount of molecular species in AR/% amount of molecular species in AS].**

| **Lipid species** | **Gu4/Gu5** | **DSY294/ DSY296** | **DSY347/ DSY289** | **DSY544/ DSY775** | **G2/G5** | **F2/F5** | **DSY290/ DSY292** | **DSY741/ DSY742** |
| --- | --- | --- | --- | --- | --- | --- | --- | --- |
| **PC 30:1** | 0.5 | 1.3 | 2.1 | - | - | 0.1 | - | 0.4 |
| **PC 32:1** | 0.6 | 1.2 | - | - | 1.2 | 0.1 | 0.8 | 0.8 |
| **PC 36:4, 36:5** | 1.3 | - | 0.4 - 0.6 | 0.3 | 1.6 | 2.5 | - | 1.2 - 1.6 |
| **PE 35:2** | 2.9 | 0.3 | - | 0.5 | 1.3 | - | 0.5 | 1.1 |
| **PE 36:4, 36:5, 36:6** | 1.4 | - | 0.5 - 0.6 | 0.08 - 0.4 | 2.1 | 1.8 - 4.5 | 0.8 | 1.1 - 1.7 |
| **PI 30:1** | 0.2 | 1.7 | 3.0 | - | - | 0.1 |  | 0.1 |
| **PI 34:1** | 0.7 | 1.2 | 1.7 | - | - | 1.7 | 1.2 | 0.7 |
| **PS 33:2** | 1.7 | 0.4 | - | 0.2 | 4.3 | 0.0 | 0.5 | - |
| **PS 35:1** | 1.4 | 0.2 | - | 0.1 | - | - | 0.5 | 1.1 |
| **PS 35:2** | 2.7 | 0.3 | - | 0.2 | - | 0.1 | 0.5 | - |
| **PG 32:0** | - | - | - | 1169.2 | n.d. in G2 | n.d. in F5 | 26.5 | 0.02 |
| **PA 36:3, 36:4, 36:5** | - | 1.1 | 1.6 | 0.2 - 0.6 | 1.7 - 2.7 | - | - | 1.1 - 1.5 |
| **MIPC 54:0;5** | 0.7 | 1.0 | 0.3 | - | - | 0.02 | - | - |
| **SE precursors (Lano-, zymo, epi-, feco- SE)** | 0.4 - 0.5 | 1.1 - 1.4 | 0.6 | - | - | 0.1 | 2.5 | - |
| **SE end products (Ergostatetraenoland ergosterol ester)** | - | 0.7 | 0.6 | - | 2.4 - 4.9 | 3.8 - 6.7 | 3.2 | - |

**‘n.d.’ means that this molecular species was not detected.**
